# Dermatologic Conditions Associated With Various Types of Popular Nail Cosmetics: A Systematic Review of Existing Literature and Future Recommendations

**DOI:** 10.1111/jocd.70519

**Published:** 2025-10-21

**Authors:** Kiran Javaid, Sonam Mistry, Madeline Tchack, Noah Musolff, Bassem Rafiq, Babar Rao

**Affiliations:** ^1^ Rowan‐Virtua School of Osteopathic Medicine Stratford New Jersey USA; ^2^ Rutgers Robert Wood Johnson Medical School Piscataway New Jersey USA; ^3^ Center for Dermatology Rutgers Robert Wood Johnson Medical School Somerset New Jersey USA; ^4^ Rao Dermatology Atlantic Highlands New Jersey USA; ^5^ Department of Dermatology Weill Cornell Medicine New York New York USA

**Keywords:** adverse manicure outcomes, allergic contact dermatitis, manicures, nail cosmetics

## Abstract

**Introduction:**

Enthusiasm for manicures is at a high amongst young populations and has led to a growth in nail cosmetology in the last decade. Reviewing the known nail and cutaneous adverse outcomes associated with manicures can improve counseling for safe usage.

**Aims:**

To provide a comprehensive review of cutaneous disorders reported with various types of nail cosmetics, to allow consumers to make informed decisions when choosing a manicure type. In addition, this article promotes cognizance amongst dermatologists of potential causes of dermatologic conditions associated with nail cosmetics.

**Methods:**

A literature review of articles from January 2014 to December 2025 was conducted on the PubMed database. A combination including, but not limited to, the following terms was utilized: nails, cosmetics, nail disease, adverse outcomes, or pedicure.

**Results:**

The most common reported adverse outcomes of gel manicures include psoriasiform onychodystrophy, pterygium inversum unguis (PIU), allergic contact dermatitis (ACD), onycholysis, paronychia, pseudo‐psoriatic nails, and onychomycosis. The most commonly reported adverse outcomes of acrylic manicures include worn‐down nail syndrome, pseudo‐psoriatic nails, ACD, onychomycosis, onycholysis, periungual eczema, and pseudo‐psoriatic allergic nail dystrophy. Risk factors for adverse outcomes include using ultraviolet (UV) light, at‐home manicures, acrylates, and isocyanates.

**Conclusion:**

With the increased popularity of manicures, awareness of adverse outcomes associated with manicure types can improve counseling and management. Inclusion of allergens in the baseline allergy patch testing series and caution for photo‐protection in at‐risk populations should be encouraged.

## 1 Introduction

Enthusiasm for manicures is at a high amongst young populations and has led to a growth in nail cosmetology in the last decade [1, 2]. The global nail market was projected to reach $15.55 billion in 2024 as the number of nail salons has increased abundantly [1, 2]. Gel and acrylic manicures have gained popularity for their sleeker look, longer‐lasting effects, and less aftercare [3]. However, the longer‐lasting effects are due to a more extensive manicure technique using ultraviolet (UV) light to cure the polish [4]. As a result of the increased enthusiasm for manicures and more abrasive techniques for longevity, it is important to investigate adverse outcomes associated with different manicure types to improve counseling and management for these patients.

## 2 Methods

A literature review of articles from January 2014 to August 2025 was conducted on the PubMed database. A select number of search terms were utilized, including a combination of the following: [((nails) AND (cosmetics) AND (nail disease)), ((nails) AND (cosmetics)) AND (adverse outcomes), (manicure) OR (pedicure)]. These yielded 277, 267, and 175 results respectively. After primary and secondary exclusion criteria were applied and duplicates removed, a total of 211 results were identified. Inclusion and exclusion criteria are listed in Table 1. After screening for relevance using titles and abstracts, a total of 48 articles were included in this analysis. The data extracted included study type, sample size, manicure type, manicure procedure, and adverse outcomes/disorders.

**TABLE 1:** Inclusion and exclusion criteria of studies.

Primary inclusion and exclusion criteria	
Inclusion	Exclusion
Articles written after 2014	Articles discussing noncutaneous effects of nail cosmetics
Case reports and studies conducted in humans	Studies not specifying specific manicure related outcomes
Articles written in English	Systematic reviews
Full‐text articles	Articles discussing pedicures exclusively

After a second targeted search was conducted using the following terms: (((“Isocyanates”[Mesh]) OR “Acrylates”[Mesh]) OR “Methacrylates”[Mesh]) AND “Nail Diseases”[Mesh], 56 additional results were identified. Ten met inclusion criteria, leading to a total of 58 articles in this analysis.

## 3 Results

The results of the study are summarized in Tables 2, 3, 4, 5.

**TABLE 2:** Overview of primary risk factors and associated outcomes of gel manicure‐related studies.

Summary of studies—Gel manicures			
Study	Dermatologic diagnosis	Description of study	Risk factors
[5]	Pterygium inversum unguis	Type: Retrospective and prospective case study No. of patients: 17 Description of patient: Female patients aged 22–57 with current use of gel polish and complaints of growth of the “skin” under the fingernails and pain when cutting or filing the nails	
[6]	Self‐reported symptoms suggesting allergic contact dermatitis (ACD)	Type: Survey No. of patients: 2118 Description of patient: Female, age range 12–60 years	– Acrylates in gel polish used
[7]	Fingertip and periungual dermatitis	Type: Case study No. of patients: 1 Description of patient: 40‐year‐old atopic, non diabetic female	– (Meth)acrylate monomers in gel polish home manicure
[4]	– Fingertip dermatitis – ACD – Onycholysis – Paronychia – Thin, brittle nails – Eczema on the lips, throat, and around the eyes	Type: No. of patients: 8 Description of patient: Patients who had reported severe skin reactions after using the specific gel polish	– (Meth)acrylate monomersin gel polish – At‐home manicure
[8]	– Chronic urticaria – ACD	Type: case study No. of patients: 1 Description of patient: 50‐year‐old woman	– Acrylates or methacrylates in gel polish
[9]	– Pseudo‐psoriatic nails – ACD – Onychomycosis – Onycholysis	Type: Case studies No. of patients: 3 Description of patients: 30‐, 45‐, 38‐year‐old females	– Acrylates or methacrylates in gel manicure
[10]	Chronic palmar eczema without nail/finger involvement	Type: Case studies No. of patients: 2, one related to gel manicure Description of patients: 26‐year‐old nonatopic woman	– (Meth)acrylate monomersin gel polish
[11]	Pseudo‐psoriatic Allergic nail dystrophy	Type: Case study No. of patients: 1 Description of patients: 38‐year‐old female	– (Meth)acrylate monomers in gel polish
[12]	Pseudo‐psoriatic nails	Type: Case study No. of patients: unspecified Description of patients: unspecified	– Acrylates in gel polish
[13]	Porphyria cutanea tarda	Type: Case study No. of patients: 1 Description of patients: 29‐year‐old female	– UV light in curing process
[14]	ACD	Type: Case study No. of patients: 1 Description of patients: 14‐year‐old female	– Acrylates in gel polish used on clients – At‐home manicure
[15]	ACD	Type: Case study No. of patients: 1 Description of patients: 14‐year‐old female	– (Meth)acrylate monomers in gel polish – At‐home manicure
[16]	Eyelid dermatitis (ACD)	Type: Case study No. of patients: 1 Description of patients: 45‐year‐old female	– Acrylates in gel polish
[17]	Anigoedema of the lips and eyelids	Type: Case study No. of patients: 1 Description of patients: 22‐year‐old female	– Acrylates – Isocyanates – At‐home manicure
[18]	Recurrent cheilitis and lip edema	Type: Case study No. of patients: 1 Description of patients: 49‐year‐old female	– Methacrylates
[19]	Pincer nail deformity, yellowish chromonychia, numerous nail pitting, pseudo leukonychia and trachyonychia	Type: Case study No. of patients: 1 Description of patients: 61‐year‐old female	
[20]	Solar urticaria	Type: Case study No. of patients: 1 Description of patients: 61‐year‐old female	– UV lamps

**TABLE 3:** Overview of primary risk factors and associated outcomes of acrylic manicure‐related studies.

Summary of studies—Acrylic manicures			
Study	Dermatologic diagnosis	Description of study	Risk factors
[21]	Worn down nail syndrome	Type: Case study No. of patients: 1 Description of patients: 24‐year‐old female	
[9]	– Pseudo‐psoriatic nails – ACD – Onychomycosis – Onycholysis	Type: Case studies No. of patients: 3 Description of patients: 30‐, 45‐, 38‐year‐old females	– Acrylates in acrylic manicure
[10]	Periungual eczema	Type: Case studies No. of patients: 2, one related to acrylic manicure Description of patients: 41‐year‐old nonatopic woman	– HEMA in acrylic polish used – At‐home manicure
[11]	Pseudo‐psoriatic allergic nail dystrophy	Type: Case study No. of patients: 1 Description of patients: 38‐year‐old female	– HEMA in acrylic manicure
[22]	Psoriasiform acral dermatitis (PAD) (form of ACD)	Type: Case study No. of patients: 1 Description of patients: 51‐year‐old female	– Acrylates in acrylic manicure
[23]	ACD	Type: Case study No. of patients: 1 Description of patients: 41‐year‐old female	– Acrylates in acrylic manicure – At‐home manicure

**TABLE 4:** Overview of primary risk factors and associated outcomes of miscellaneous manicure‐related studies.

Summary of studies—Miscellaneous manicure type				
Study	Type of manicure	Dermatologic diagnosis	Description of study	Risk factors
[24]	Russian manicure/pedicure	Acute Paronychia and Onychomadesis	Type: Case report No. of patients: 1 Description of patients: 20‐year‐old female	– Cuticle manipulation (removal) – Water immersion – Classic nail polish
[25]	Dip manicure	– Dyshidroticlike Contact Dermatitis – Paronychia	Type: Case report No. of patients: 1 Description of patients: 58‐year‐old female	– HEMA in dip powder
[26]	Hybrid manicure	ACD	Type: Case report No. of patients: 1 Description of patients: 33‐year‐old female	– Methacrylates in shellac polish patient used
[27]	Hybrid manicure	Nail plate structure changes—changes in the height of the nails – Dimples – Disap‐pearance of the natural furrowing of the nail – Protrusions as the remains of the light curable preparation	Type: single center, open label trial No. of patients: 83 Description of patients: 18–50‐year‐old females	– Preparations applied to the nails, acetone, or filing the nail plate
[28]	Shellac manicure	ACD	Type: Case reports No. of patients: 4 Description of patients: 23‐year‐old beautician, 35‐year‐old beautician, 20‐year‐old beauty therapist, 24‐year‐old nurse	– Methacrylates in Shellac polish patients used – At home use
[29]	Nail enamel	– Decreased nail health: increase in the nail depth and a decrease in its density – Nail lesions	Type: single center, open label trial No. of patients: 83 Description of patients: 18–50‐year‐old females	– Acetone, ethyl acetate, and ethyl lactate in nail enamel
[30]	At‐home press on nails	ACD	Type: Case report No. of patients: 1 Description of patients: 13 year old female	– Acrylates in nail glue used – At‐home manicure – Patient's age
[31]	Formaldehyde in nail hardeners	ACD and nail damage mimicking psoriasis	Type: Case report No. of patients: 2 Description of patients: 39‐year‐old female, 49 year‐old female,	– Formaldehyde

**TABLE 5:** Overview of primary risk factors and associated outcomes of overall manicure procedure‐related studies.

Summary of studies—Overall manicure procedure				
Study	Procedural step	Dermatologic diagnosis	Description of study	Risk factors
[32]	UV light	– Multiple actinic keratoses on dorsal hands – multiple squamous cell carcinomas on dorsal hands	Type: Case study No. of patients: 1 Description of patients: 45‐year‐old female with history of renal transplant	– Two times renal transplant recipient – Immunosuppressive therapy: tacrolimus, mycophenolate mofetil, and prednisolone – Monthly manicures for 20 with unregulated ultraviolet radiation A (UVA)
[33]		Periungual cutaneous lupus erythematosus lesions	Type: Case study No. of patients: 2 Description of patients: Patient 1: 35‐year‐old South Asian female with a history of SLE and DLE diagnosed 12 years ago Patient 2: 40‐year‐old African American female with a history of SLE and DLE	– SLE and DLE – 250 mg of chloroquine three times per week, 0.6 mg colchicine daily, 3000 mg of MMF daily and clobetasol 0.05% ointment. – 100 mg of HCQ daily, 100 mg of AZA daily and 6 mg of methylprednisolone daily, and topical steroids including pimecrolimus 1% cream, triamcinolone 0.1% ointment and clobetasol 0.05% ointment. – Both patients had been receiving manicures for several years approximately once every 1–2 months
[7]	Nail glue and artificial nail tip	ACD	Type: Case study No. of patients: 1 Description of patient: 40‐year‐old atopic, non diabetic female	– Presence of Isobornyl acrylate (IBOA) was listed in glue used—Saviland Solid Nail Glue Gel, a UV‐cured adhesive – At‐home manicure
[34]		Full thickness burns	Type: Case study No. of patients: 3 Description of patient: 15‐, 11‐, 16–year‐old females	– Cyanoacrylate in nail glue – Exothermic reaction between nail glue and cotton clothing

## 4 Discussion

Nail manicures offer a wide range of application methods, costs, and styles, with over 10 distinct forms available in the cosmetic industry. Acrylic nails are a type of artificial nail enhancement created from a mixture of both ethyl methacrylate (EMA) liquid and powder acrylates that harden with air exposure and do not require UV photocuring [35]. Acrylics are also referred to as “sculpted” or “porcelain” nails, typically sculpted and shaped over bare nails with artificial tips. Gel polish is an acrylic‐based nail product composed of polymerization photoinitiators and (meth)acrylate monomers—such as 2‐hydroxyethyl methacrylate (HEMA), methacrylates, and acrylates—which belong to the (meth)acrylate family of esters. Three layers (base, color, and top coat) are cured under UV/LED light, causing a free‐radical reaction that hardens the polish [5].

Acrylic and gel manicures are reported to be the most common kinds of manicures [36] and were also linked to the highest incidence of adverse outcomes (see Tables 2 and 3).

### 4.1 Adverse Outcomes

The most common adverse outcome associated with gel manicures was allergic contact dermatitis (ACD). ACD involving nails typically presents with periungual erythema, swelling, onycholysis, and nail plate dystrophy, often accompanied by pruritus or tenderness. Nearly half of all articles discussing gel manicures reported ACD as an adverse outcome. Similarly, 60% of articles discussing acrylic nails reported ACD as an adverse outcome. Interestingly, the conglomerate of studies suggests that the presence of acrylates may be the primary risk factor for adverse outcomes, especially ACD, for both acrylic and gel manicures [37, 38, 39, 40, 41, 42]. In a review of ingredients in 394 nail products, 2‐hydroxyethylmethacrylate (HEMA), a type of acrylate, was listed in approximately 60% of products [7]. Steunebrink et al. found that in 8.5 years, 67 patients were diagnosed with ACD from nail cosmetics and 97% of subjects had a positive patch test to HEMA [37].

As the prevalence of (meth)acrylate allergy continues to rise, it is important to highlight that nail technicians and beauticians are substantially more affected. In a 7‐year study on methacrylates and acrylates exposure, Ramos et al. found that nail beauticians were the most affected group, representing 80% of all occupational cases [43]. In a study conducted specifically on nail salon workers, presentations of ACD included erythematous dermatitis of the dorsal hands, palms, forearms, fingertip fissures, periorbital regions, cheeks, posterior ears, neck, sacral area, lateral thighs, and dorsa of the feet [44].

Of note, ACD due to acrylates tends to occur at the site of application [16] and less commonly occurs in distant areas such as the face and eyelids. Moreira et al. found that distant ACD can be explained by hand transportation or airborne dissemination of the allergen, a finding corroborated by Kieć et al. Ocular symptoms reported by participants in Kieć et al.'s study included itching, redness, and tearing [45]. These findings in conjunction suggest that exposure to nail cosmetics should be considered when evaluating eyelid dermatitis.

Another common clinical pattern observed is pseudo‐psoriatic nails, particularly in individuals wearing acrylic products. The presenting symptoms are similar to those seen in psoriatic nails, such as onycholysis and significant subungual hyperkeratosis [9]. Pseudo‐psoriatic nails are believed to be caused by the traumatic removal of firmly attached acrylic nails, though they have been seen prior to such removal processes [9].

Although there are similarities between the adverse outcomes of gel and acrylic manicures, some outcomes are more so associated with one type over the other. Worn down nail syndrome (WDNS) is described as a triangular area of nail thinning with pinpoint hemorrhages and dilated capillaries [21]. Acrylic nails often require abrasive mechanical techniques to remove, which can lead to WDNS. Dickison and Smith [8] were the first to identify chronic urticaria due to gel nails. All 17 subjects developed pterygium inversum unguis (PIU), presenting as adhesion of the nail bed and hyponychium to the ventral nail plate, after 2–5 years of gel polish application [5]. They also suggest that PIU secondary to gel manicure is frequently overlooked but not uncommon.

Allergic contact dermatitis mimicking angioedema was also identified as a potential outcome. In one case of recurrent nonpruritic chelitis and angioedema, patch testing with the European baseline series reported reactions to many acrylates and methacrylates [18]. Cessation of regular manicures resulted in rapid resolution. In another reported case, inspection of gel nail products revealed fragrances, (meth)acrylates, and polyurethane (isocyanates) [17]. These cases highlight important considerations of the non‐occupational and cosmetic causes of angioedema.

### 4.2 Effect of At‐Home Manicures

At‐home manicures are associated with a variety of adverse effects, but most prominently ACD and burns, especially in the absence of an expert beautician [46]. A cross‐sectional survey of 199 individuals found that home‐acrylic‐nail‐kit use was associated with earlier development of skin reactions and more frequent nail damage than professional acrylic manicures [47]. They also learned that 68% of participants learned about at‐home nail kits through social media, and 74% received training through online tutorials. 83% of home nail kit users experienced skin reactions for the first time after their first time using home kits.

Degrees of ACD may be higher amongst users of home nail kits, possibly due to improper curing techniques with incorrect wavelengths that prevent the nails from properly hardening [10, 14, 48]. Kjeldsen et al. demonstrate isolated palmar eczema from HEMA contamination, possibly from incomplete curing of the nail product, as different types of acrylic products polymerize at varying UV light wavelengths.

Home products also allow children and adolescents easy accessibility to chemicals and allergens in nail products. Kelemen et al. highlight several cases of adolescents experimenting with beauty products and using cyanoacrylates commonly used in nail glues (Table 5). However, ill knowledge of the dangerous exothermic reaction between cotton fibers and nail glue led to several cases of full‐thickness thermal burns while applying artificial nails. A literature review on burn injuries related to cyanoacrylate‐based nail glue reported 15 cases associated with partial to full‐thickness burns following spillage of glue at home [49]. Thirteen reported cases were children, with the youngest patient being 5 months old [49]. Tramontana et al. also concluded that self‐application by untrained and younger patients leads to a higher risk of gel containing acrylate monomers overflowing from the nail plate and contacting the periungual skin, enhancing sensitization to acrylates [14]. Future studies could further explore other outcomes associated with at‐home manicures.

Notable, however, Wilkinson et al. reported that despite the prevailing sentiment, methacrylate‐containing salon products may still induce sensitization. Of 38, 36 (94.7%) consumers with contact allergy to (meth)acrylate related to nail cosmetics had these products applied exclusively at a salon [50]. This highlights that caution should be exercised in both home usage and salon usage.

### 4.3 Effect of Prior Health Conditions

Prior health conditions of individuals getting manicures requiring UV light is a pertinent risk factor for poorer outcomes. A case study of a 45‐year‐old woman on immunosuppressive therapy presented for skin cancer surveillance with actinic keratoses and skin grafts on her hands following previous squamous cell carcinomas. It was found that she had been using unregulated UVA‐emitting lamps for monthly manicures over the past 20 years. Although no definitive link to her skin cancers currently exists, caution with UVA exposure in immunosuppressed patients is encouraged [32].

In addition, UV light, particularly UVA, has an established correlation with the incidence and progression of both systemic lupus erythematosus (SLE) and cutaneous lupus erythematosus (cLE). Two patients with SLE and discoid lupus erythematosus (DLE) developed periungual cLE lesions after using gel manicures [33]. Both cases resolved over time, suggesting that UV exposure from nail lamps can exacerbate cLE.

Another study outlined a 29‐year‐old woman who developed blisters on the dorsal surfaces of her hands and digits after a manicure involving UV light exposure at a nail salon [13]. Initially, she was diagnosed with hand dermatitis, but biopsy later confirmed porphyria cutanea tarda (PCT). This case highlights the importance of considering manicure types in the etiology of blistering conditions like PCT, particularly when phototherapy has worsened the condition.

### 4.4 Other Types of Manicures

Other popular, but harmful, manicure types include Russian manicures, dip manicures, shellac manicures, press‐on nails, etc. (Table 5). Russian manicures file and remove the cuticle, an important part of the nail unit, and serve as a barrier to prevent infection and paronychia [24].

Another popular type is a hybrid manicure, which involves chemical exposure, UV radiation, and mechanical abrasion. This decreases nail strength by compromising the integrity of keratin [51]. Hybrid manicures that use daylight instead of UV or LED to cure gel manicures also lead to hand dermatitis and ACD [52]. (Meth)acrylate monomers are present in hybrid nail lacquers, and this manicure type harbors the same risks as UV and LED‐cured manicures [26, 52].

Classic manicures that do not use curing techniques historically contain allergens such as toluene, formaldehyde, and dibutyl phthalate (DBP). A recent study shows that these allergens are falsely reported to be eliminated from nail polish products, known as “3‐free” products, and that chemical exposures to formaldehyde and toluene are still apparent in classic nail lacquers [53]. It is unclear why these products are falsely reported as “3‐free,” but consideration of these allergenic irritants should be given to patients with nail ACD who get classic manicures.

In addition to these allergens, we highlight the case of nail dystrophy mimicking psoriatic disease in a patient due to copolymer containing nail varnish [54]. Although copolymers have been used in nail products as alternatives to the historically common allergen tosylamide formaldehyde resin (TSFR), they themselves are potential contact allergens. Coe et al. illustrate these findings in a case report showing positive patch test results to adipic acid–neopentyl glycol–trimellitic anhydride copolymer (AA) and phthalic anhydride–trimellitic anhydride–glycols copolymer (PA) [54].

### 4.5 Considerations for Dermatologists

With the increased popularity of manicures, awareness of possible complications of different types of manicures prompts appropriate patient education. Recent recommendations include restriction of the application of long‐lasting nail polishes to professionals and advising against home usage [42]. Avoidance of contact with acrylate‐containing products resulted in completely clearing dermatitis in 80% of patients, supporting the inclusion of HEMA allergen in baseline allergy testing [37, 55, 56]. This could prove beneficial in cases of unclear cheilitis and angioedema as well [18].

Though carcinogenic potential is low [57, 58, 59, 60], caution should be advised for all manicure types using light to cure longer‐lasting manicures (e.g., UV, LED, daylight). UV gel manicures are reported to carry additional risks for additional conditions, such as skin cancers and solar dermatoses, especially for patients with risk factors. More research to understand the etiology of this increased risk is needed [60, 61].

Until then, safeguards should be encouraged to reduce the risk of cutaneous malignancy due to manicures, such as UV‐blocking gloves or sunscreen [60]. Dermatologists should advise lupus patients on protective measures such as non‐UV drying methods or gloves and sunscreen when encountering UV light.

### 4.6 Limitations and Future Considerations

Limited data was available on the frequency of use for each type of manicure, which could be a confounding variable when comparing the frequency of associated outcomes and manicure types. Certain associations could be overstated or understated due to limited information on the frequency of manicures as well as disparities in the amount of papers available on various manicure types. In addition, a number of articles found in our search were case reports, leading to small sample sizes that could limit generalizability. Larger‐scale studies investigating the adverse effects of manicures are needed to corroborate the findings discussed herein.

Further, various steps were identified as harmful throughout the process of getting a manicure, such as improper sanitization between clients. Even with the knowledge that sharing equipment could lead to viral infections, individuals continued to share equipment [62, 63]. Future studies can also explore ways to mitigate improper sanitization techniques.

## Author Contributions

The concept of this literature review and article screening was conducted by K.J. and S.M.; K.J. performed the research. K.J. and S.M. wrote the paper; K.J., S.M., and M.T. designed the research study. Editing and revisions of the manuscript were done by K.J., S.M., M.T., N.M., B.R., and B.R. All authors have read and approved the final manuscript.

## Ethics Statement

This article is a review article of previously published literature, and no studies with human participants or animals were performed by any of the authors. Therefore, institutional review board approval and informed consent were not required.

## Consent

The authors have nothing to report.

## Conflicts of Interest

The authors declare no conflicts of interest.

## Data Availability

All data generated or analyzed during this study are included in this article. The search strategy and extracted data tables are available within the article, and more information can be obtained from the authors upon request.
